# Stigma impedes HIV prevention by stifling patient–provider communication about U = U

**DOI:** 10.1002/jia2.25559

**Published:** 2020-07-19

**Authors:** Sarah K Calabrese, Kenneth H Mayer

**Affiliations:** ^1^ Department of Psychological and Brain Sciences George Washington University Washington DC USA; ^2^ Department of Prevention and Community Health George Washington University Washington DC USA; ^3^ Beth Israel Deaconess Medical Center Harvard Medical School Boston MA USA; ^4^ The Fenway Institute Fenway Health Boston MA USA

**Keywords:** ARV, stigma, viral suppression, undetectable=untransmittable, provider

The success of HIV control strategies throughout the world depends on stakeholders’ implementation of the latest advancements in HIV science. However, healthcare providers’ adoption of recent HIV‐related scientific advancements into clinical practice has been variable. There has been a notable challenge around consistently communicating the discovery that sustained viral suppression eliminates risk of sexual transmission (undetectable = untransmittable, (U = U)) to patients. Failure to routinely incorporate U = U patient education into clinical practice is peculiar because the U = U message aligns with treatment goals. Moreover, it is providers’ professional responsibility to inform patients of treatment risks and benefits. So why aren’t these conversations happening? Although there are multiple contributing factors, including structural challenges that vary by setting (e.g. time limits on patient visits), we contend that stigma – that is, social devaluation based on one or more distinguishing characteristics [1] – could be a key reason underlying the lack of consistent U = U patient education.

Early research investigating providers’ perspectives and experiences surrounding U = U [2, 3, 4, 5] suggests several reasons why they may not be communicating about U = U with patients: lack of knowledge; disbelief (despite robust evidence demonstrating that virologic suppression prevents sexual transmission [6, 7]); and concerns about sexual risk compensation [2, 3, 4]. Providers have also expressed fear of being blamed if transmission were to occur after they had educated patients about U = U [2, 3]. When communication about viral suppression and risk does occur, some providers are inconsistent and/or unclear, continuing to use language such as “extremely low” or “negligible” (rather than “no” or “zero”) to describe transmission risk, or incorrectly qualifying U = U as applicable only in the context of condom use [2, 5]. Withholding patient education around U = U or tempering the message to prevent unwanted behaviour is not medically justifiable. Furthermore, the decision to withhold or modify U = U messaging could be influenced by stigma towards patients.

Stigma can operate at conscious (explicit) and unconscious (implicit) levels [8]. However, these two levels of stigma are not strongly correlated [8], suggesting that providers who do not consciously endorse stigma can nonetheless harbour such attitudes at an unconscious level. Implicit stigma in particular has been found to impact patient–provider interactions: Providers with higher implicit stigma verbally dominate conversations and are less patient‐centred [9], which could compromise communication around U = U.

The discretionary nature of U = U communication increases its susceptibility to stigma. At present, standards establishing patient education about U = U as part of routine care are newly emerging. In December 2019, the US government added a recommendation for universally educating patients with HIV about U = U in their antiretroviral treatment guidelines [10]. Likewise, the WHO suggested in their November 2019 HIV testing services guidelines that at the time of diagnosis, providers should educate patients that “people with HIV on [antiretroviral therapy] who achieve and maintain viral suppression cannot transmit HIV to their partners” [11]. However, in many clinical centres, standards and guidelines surrounding U = U may be absent or lack specificity, hindering routine patient education about U = U. Additionally, in such circumstances, whether, how, and whom to educate about U = U is commonly based on providers’ discretion, which is problematic because stigma is more likely to manifest in settings with ambiguous norms and/or flexible protocols [12].

It is not only the discretionary nature of U = U education as a clinical activity that renders it vulnerable to stigma; it is also the patient populations impacted and behaviours associated with HIV transmission that may be stigmatized in and of themselves. Worldwide, people living with HIV have been mistreated in healthcare, facing providers who refuse to treat or provide substandard treatment [9, 13]. This is consistent with stigmatizing attitudes towards patients with HIV that providers have endorsed, including stereotypes related to sexual irresponsibility [13]. Given the concerns related to risk compensation that providers have reported as reasons for not discussing U = U [2], these preconceived notions about patients living with HIV may reinforce existing concerns and potentiate stigma. People living with HIV who have other, intersectional marginalized statuses (e.g. men who have sex with men or people who inject drugs) may be more likely to be stereotyped as irresponsible or at risk, further exacerbating providers’ concerns about the consequences of U = U discussions and fuelling disparities.

Intersectional stigma can also compromise U = U education because U = U education requires patient–provider communication about sex, and providers are less comfortable discussing sexual behaviour with some groups (e.g. sexual minority and/or gender non‐conforming individuals) than others (e.g. heterosexual, cisgender individuals) [14]. Discomfort discussing sex with certain patients may translate to less consistent communication of the U = U message to those populations in particular. Likewise, certain stigmatized groups, such as Black American men who have sex with men, may be less comfortable initiating conversations about their sexual health with their providers because of anticipated stigma [15] and thus more reliant on providers to initiate discussions about HIV transmission during sex.

Importantly, stigma can manifest as reasoned decision making [9]. For example risk compensation concerns may be expressed as genuine consideration for patients’ wellbeing. However, such logic is likely rooted in stereotypes rather than science, as demonstrated by its inapplicability within other medical contexts. For example, educating patients about the benefits of contraceptive or erectile dysfunction medications could lead to sexual risk compensation among patients electing to take such medications, yet these benefits are nonetheless routinely communicated.

There are several strategies that may help to address existing inconsistencies and potential disparities in providers’ delivery of U = U education (Table 1). Establishing universal U = U patient education in normative guidelines, incorporating U = U into clinical education for all HIV service providers, facilitating patient–provider conversations about U = U with concrete tools, and broadening public awareness through public health messaging could all promote positive change. The latter strategy is also vital because some stigmatized groups face barriers to healthcare that prevent them from learning about U = U from providers altogether. Additional research is needed to assess the impact of provider stigma, evaluate culturally tailored interventions, and ultimately optimize U = U communication between patients and providers. Nonetheless, immediate action can and should be taken to encourage providers to routinely communicate about U = U with all of their patients and to ensure that stigma does not stifle these critical conversations.

**Table 1:** Recommended Strategies for Encouraging Universal U = U Patient Education

Strategy		Description/rationale
1	Establish universal U = U patient education in normative guidelines dictating clinical practice	Universal U = U patient education should be endorsed by federal and professional organizations throughout the world and advocated in clinical centre protocols and expectations For example, according to the US Department of Health and Human Services 2019 guidelines, “All persons with HIV should be informed that maintaining a plasma HIV RNA (viral load) <200 copies/mL… prevents sexual transmission of HIV to their partners” [10] Establishing such a standard reinforces U = U patient education as a professional responsibility and designates failure to communicate U = U with patients, even if not an intentional omission, as substandard care
2	Incorporate U = U into clinical education for all HIV service providers	U = U should be incorporated within clinical education at all levels, including medical and nursing school curricula, board certification exams, continuing education, and required clinical trainings Providers should be informed about: the scientific evidence for U = U, the medical and psychosocial implications of U = U for patients, the importance of a universalized vs. selective approach to educating patients about U = U, and why fears of risk compensation or blame are not medically justifiable reasons to withhold information about U = U Widespread U = U clinical education across HIV service provider disciplines is needed because patient education is a shared responsibility across HIV service providers, and a given patient may come into contact with some types of HIV service providers and not others Provider education about U = U is essential because lack of knowledge and disbelief are among the identified reasons for providers' failure to inform their patients about U = U [2, 4]
3	Facilitate patient–provider conversations about U = U with concrete tools	Providers can be offered empirically supported, scripted language to help explain the concept of U = U Prompts can be used to cue conversations about U = U, such as pairing pop‐up reminders with viral load laboratory results within electronic medical record systems Informational pamphlets, closed‐circuit waiting room videos, and other patient‐targeted education materials can further stimulate and reinforce patient–provider conversations about U = U
4	Broaden public awareness through public health messaging	Public education can encourage patient‐initiated conversations among individuals living with HIV who are already in care Increasing public knowledge about U = U may promote healthcare‐seeking among individuals living with HIV who are undiagnosed or untreated; new patients present new opportunities for patient–provider communication about U = U Beyond healthcare implications, public education is also vital because certain stigmatized groups, particularly those facing intersectional forms of stigma, may face barriers to healthcare that prevent them from learning about U = U from providers altogether

## COMPETING INTERESTS

SKC received partial support from Gilead Sciences to attend a research conference. KHM has conducted research with unrestricted project support from Gilead Sciences and ViiV Healthcare.

## AUTHORS’ CONTRIBUTIONS

SKC led the conceptualization and writing of the Viewpoint with significant input from KHM. Both authors have read and approved the final manuscript.

## ACKNOWLEDGEMENTS

None declared.

### FUNDING

None declared.

### DISCLAIMER

None declared.
